# Valedictory Address

**Published:** 1859-04

**Authors:** 


### VALEDICTORY ADDRESS.

We have received the valedictory address to the graduating class of the Philadelpha college of medicine, at the annual commencement, held March 2d, 1859, by J. Aitkin Meigs, M. D., Professor of the Institutes of Medicine, one of the Consulting Physicians to the Philadelphia Hospital, &c.

We have read this address with *pleasure* and *profit*, which is saying much more than can be usually said of introductory and valedictory medical addresses.

The responsibilities, difficulties, pleasures, and rewards of the profession, are set forth in an unusually interesting and forcible way.

